# Multifactorial determinants of NK cell repertoire organization: insights into age, sex, KIR genotype, HLA typing, and CMV influence

**DOI:** 10.3389/fimmu.2024.1389358

**Published:** 2024-04-26

**Authors:** Enora Ferron, Gaëlle David, Catherine Willem, Nolwenn Legrand, Perla Salameh, Laetitia Anquetil, Alexandre Walencik, Ketevan Gendzekhadze, Katia Gagne, Christelle Retière

**Affiliations:** ^1^ Etablissement Français du Sang, Nantes, France; ^2^ INSERM UMR1307, CNRS UMR 6075, CRCI2NA, team 12, Nantes, France; ^3^ LabEx IGO “Immunotherapy, Graft, Oncology”, Nantes, France; ^4^ Laboratoire d’histocompatibilité de l’Etablissement Français du Sang de Centre-Pays de la Loire, Nantes, France; ^5^ Department of Hematology and Hematopoietic Stem cell Transplantation (HCT), Human Leukocyte Antigen (HLA) Laboratory, City of Hope, Medical Center, Duarte, CA, United States; ^6^ LabEx Transplantex, Université de Strasbourg, Strasbourg, France

**Keywords:** KIR, HLA, NK cells, repertoire, CMV, CD57

## Abstract

**Introduction:**

Polymorphisms in the KIR and HLA genes contribute to the diversity of the NK cell repertoire. Extrinsic factors also play a role in modifying this repertoire. The best example is cytomegalovirus, which promotes the expansion of memory-like NK cells. However, the mechanisms governing this phenotypic structure are poorly understood. Furthermore, the influence of age and sex has been understudied.

**Methods:**

In this study, we examined these parameters in a cohort of 200 healthy volunteer blood donors, focusing on the major inhibitory KIR receptors and CD94/NKG2A, as well as the differentiation marker CD57 and the memory-like population marker NKG2C. Flow cytometry and two joint analyses, unsupervised and semi-supervised, helped define the impact of various intrinsic and extrinsic markers on the phenotypic structure of the NK cell repertoire.

**Results:**

In the KIR NK cell compartment, the KIR3DL1 gene is crucial, as unexpressed alleles lead to a repertoire dominated by KIR2D interacting only with HLA-C ligands, whereas an expressed KIR3DL1 gene allows for a greater diversity of NK cell subpopulations interacting with all HLA class I ligands. KIR2DL2 subsequently favors the KIR2D NK cell repertoire specific to C1/C2 ligands, whereas its absence promotes the expression of KIR2DL1 specific to the C2 ligand. The C2C2Bw4+ environment, marked by strong -21T motifs, favors the expansion of the NK cell population expressing only CD57, whereas the absence of HLA-A3/A11 ligands favors the population expressing only NKG2A, a population highly represented within the repertoire. The AA KIR genotype favors NK cell populations without KIR and NKG2A receptors, whereas the KIR B+ genotypes favor populations expressing KIR and NKG2A. Interestingly, we showed that women have a repertoire enriched in CD57- NK cell populations, while men have more CD57+ NK cell subpopulations.

**Discussion:**

Overall, our data demonstrate that the phenotypic structure of the NK cell repertoire follows well-defined genetic rules and that immunological history, sex, and age contribute to shaping this NK cell diversity. These elements can contribute to the better selection of hematopoietic stem cell donors and the definition of allogeneic NK cells for cell engineering in NK cell-based immunotherapy approaches.cters are displayed correctly.

## Introduction

NK cells are the main effector lymphocytes of innate immunity and play a pivotal role in controlling infections and eliminating cancer cells. Due to their wide range of activating and inhibitory receptors, NK cells can sense phenotypic alterations. The sum of activating and inhibitory inputs defines the global NK cell response. Human leukocyte antigen (HLA) constitutes the identity of nucleated cells, and NK cells discriminate between self and non-self by sensing HLA class I expression via inhibitory receptors, such as killer cell immunoglobulin-like receptors (KIR) and the CD94/NKG2A heterodimer. KIR recognizes HLA class I molecules (HLA-A, -B, and -C), whereas CD94/NKG2A recognizes the nonclassical HLA-E molecule, which contains peptides derived from the leader sequence of HLA class I molecules (1). Although the HLA and KIR genes segregate independently on different chromosomes, functional KIR–HLA interactions that mediate innate NK cell immunity drive their coevolution in an ever-changing environment of pathogen exposure and human reproduction (2, 3).

Similar to their ligands, KIR genes exhibit significant allelic polymorphisms, along with haplotype variation and variable gene copy numbers and content (4, 5). Two haplotypes have been identified based on the KIR gene content (6). The KIR A haplotype comprises only 7 fixed KIR genes (3DL3, 2DL3, 2DL1, 2DL4, 3DL1, 2DS4, and 3DL2), including the sole-activating gene KIR2DS4. The KIR B haplotypes, containing variable KIR gene content (8 to 14 genes), include one or more of the B-specific KIR genes, such as 2DL2, 2DL5, 3DS1, 2DS1, 2DS2, 2DS3, and 2DS5. Based on the combination of the KIR A and B haplotypes, 660 different KIR genotypes (AA and Bx) have been described in 25,116 individuals from 192 populations (http://www.allelefrequencies.net/). The KIR locus comprises centromeric (Cen) and telomeric (Tel) gene content motifs. Due to this organizational pattern, a robust linkage among different KIR genes has been observed, with variations across populations. Inhibitory KIR genes are more polymorphic than their activating forms. KIR2DL1 and KIR2DL2/3 are found in the majority of KIR genotypes; however, some alleles are associated with specific A or B haplotypes. For example, the KIR2DL1*003 allotype is associated with the Cen A motif and confers the highest NK cell frequency, expression level, and strength of KIR–HLA-C interactions compared to the KIR2DL1*002 and *004 allotypes associated with Cen B motifs. The KIR2DL2*001 and *003 allotypes negatively affect the frequency of KIR2DL1^+^ and KIR2DL3^+^ NK cells (7). KIR3DL1 is highly polymorphic, with 307 alleles, as reported in the latest IPD KIR database. The allelic KIR3DL1 polymorphism affects the expression level of KIR3DL1 on the cell surface, with null (KIR3DL1*004), low (KIR3DL1*005), or high (KIR3DL1*001) expression. These allelic differences affect the function of KIR3DL1^+^ NK cells and may directly influence hematopoietic stem cell outcomes (8) or diseases, such as AIDS progression and the onset of psoriasis (9). The proportion of individuals without KIR3DL1 expression is significant, with almost 20% of European populations (8).

HLA-C molecules are the main KIR ligands and form two groups, C1 and C2, based on the pairs of dimorphic amino acids at positions 77 and 80, respectively. The C1 ligands (Ser^77^ and Asn^80^) are recognized by KIR2DL2/3, whereas the C2 ligands (Asn^77^ and Lys^80^) are recognized by KIR2DL1 (10, 11). KIR2DL2 and KIR2DL3 are more restricted by peptide sequence than KIR2DL1 for binding to HLA-C, whereas C2 recognition by KIR2DL1 is unique in being largely peptide agnostic (12). Thus, this peptide specificity balances KIR binding affinity, as KIR2DL2/3 exhibits a large spectrum of HLA-C recognition in contrast to KIR2DL1, which recognizes only C2 ligands (13). KIR3DL1 recognizes HLA-A and -B molecules harboring serologic Bw4 motifs with notable exceptions (e.g. A*25:01, B*13:01). KIR3DL2 recognizes an epitope shared by HLA-A3 and HLA-A11 allotypes. This recognition appears to be peptide specific. HLA-C molecules are ligands of KIR2DL1/2/3 receptors, which are expressed by almost everyone. However, KIR3DL1 and KIR3DL2 are not universally expressed, and HLA-A and -B ligands are not considered primary KIR ligands. Activating KIR2DS1 recognizes C2 ligands as its inhibitory homolog, but it is functional only in C2^-^ individuals (14, 15), whereas KIR2DS2 recognizes C1 ligands regardless of the nature of the HLA-C environment (13). Although innate NK cells are not antigen specific, they are functionally educated to recognize HLA ligands via inhibitory KIR (16). This recognition enables NK cells to identify altered HLA ligand expression in abnormal cells, and significantly contributes to the activation of NK cell cytotoxicity. Uneducated NK cells are not eliminated as T lymphocytes during thymic selection but persist in a hyporesponsive state. This educational mechanism can be modulated by various physiological factors, including KIR and HLA ligands, as well as cytokines. Additionally, pathological factors, such as viral infections, also play a role (17). This is a dynamic process that evolves over time and adapts to the NK cell environment.

In contrast to T lymphocytes, the diversity of the NK cell repertoire does not depend on receptor gene recombination. Natural variations in innate immune cell parameters are preferentially driven by genetic factors (18). This is based on the functional interaction of KIR–HLA pairs, which are the most polymorphic in humans (19), and relies on a high number of combinations of different NK receptor types. Interestingly, Horowitz et al. estimated 6,000–30,000 phenotypic populations within an individual and more than 100,000 phenotypes in an entire donor panel using multiparametric mass cytometry to analyze the expression of 28 NK cell surface markers (20). Thus, stochastic expression of KIR and heterogeneously expressed activating/inhibitory receptors contribute to the intra/inter-individual diversity of the NK cell repertoire (21).

Interestingly, immune history drives the NK cell repertoire. Numerous studies have shown that interactions between HLA and inhibitory KIR can drive HIV-1-mediated immune evasion (22). Additionally, HIV-1-associated changes in the KIR repertoire of NK cells are predetermined by host *KIR2DL/HLA-C* genotypes (23). HIV appears to shape the KIR NK cell repertoire by increasing the number of KIR2D NK cell compartments, particularly KIR2DL1^+^ NK cell subsets, in C2C2 individuals. Adaptive NK cells, identified in 40% of cytomegalovirus (CMV)-seropositive individuals, are a terminally differentiated NK cell subset driven by CMV infection (24, 25). Memory NK cells are similar to memory CD8^+^ T-cells and possess common epigenetic signatures (26).

Numerous studies have established key parameters influencing the organization of the NK cell repertoire; however, the articulation of these factors and the rules governing this structure remain poorly understood. In this study, we examined all of these parameters (KIR and HLA genetics, age, sex, and CMV status) in a cohort of 200 healthy volunteer blood donors, focusing on the NK cell education-engaged inhibitory receptors KIR (KIR2DL1, KIR2DL2/3, and KIR3DL1) and CD94/NKG2A, as well as the differentiation marker CD57 and the memory-like population marker NKG2C.

## Materials and methods

### Blood donor samples

Peripheral blood mononuclear cells (PBMCs) were isolated as previously described (15). All blood donors were recruited at the Blood Transfusion Center (EFS, Nantes, France), and informed consent was obtained from all individuals. Authorization for the preparation and conservation of biocollections (AC-2021-4397) was provided by the French Research Minister. Cells were cultured in RPMI 1640 medium (Gibco, Paisley, Scotland, UK) containing glutamine (Gibco) and penicillin-streptomycin (Gibco) and supplemented with 10% fetal bovine serum (Gibco).

### HLA and KIR genotyping

HLA class I allele assignment was performed for all blood (n=200) and registered volunteer bone marrow donors (n=12,546) as previously described (27). Genetic typing of KIR was performed on all blood donors using the KIR multiplex PCR-SSP method, as previously described (28), with primers provided by Dr. Ketevan Gendzekhadze. The presence or absence of KIR2DL1, 2DL2, 2DL3, 2DL5, 3DL1, 2DS1, 2DS2, 2DS3, 2DS4/1D, 2DS5, and 3DS1 was determined. Donor KIR genotypes were determined based on the presence or absence of activating KIR. Thus, a KIR AA genotype was defined by the presence of only KIR2DS4 as an activating KIR gene, while a KIR B^+^ genotype was defined by the presence of several activating KIR genes (29). KIR ligands, such as HLA-A3/A11, Bw4 (HLA-A and/or HLA-B), C1, and C2, were identified in all blood donors based on allelic HLA-A, -B, and -C typing. HLA-E binds to a conserved peptide cleaved from the leader sequences of HLA-A, -B, or -C. While HLA-A and HLA-C supply a peptide leading to HLA-E binding, methionine/threonine dimorphism at position -21 of the leader sequence divides HLA-B allotypes into two groups: the first group with -21T, which does not supply HLA-E-binding peptides, and the second group with -21M, which does. Thus, we distributed all blood donors into three groups: -21T, -21M, and -21T/M.

### KIR allele typing

To assign KIR alleles in all volunteer blood donors, KIR genes were captured by long-range PCR and subjected to sequencing on a MiSeq sequencer (Illumina, San Diego, CA, USA) after library preparation, as previously reported (30). KIR allele assignment was performed using the Profiler software version 2.24, developed by M. Alizadeh (Research Laboratory, Blood Bank, Rennes, France). An updated KIR allele library available in the IPD-KIR 2.8 database was implemented in Profiler.

### Cell phenotypic analysis by flow cytometry

The NK cell phenotype from healthy blood donors was determined by seven-color multiparameter flow cytometry on PBMCs using the following mouse anti-human mAbs: anti-NKp46-PE (9E2, Biolegend, San Diego, CA, USA), anti-KIR2DL1/S1-FITC (VPB6, Miltenyi Biotec, Paris, France), anti-KIR2DL2/3/S2-PerCP-Cy5.5 (GL183, Beckman Coulter, Immunotech, Marseilles, France), anti-KIR3DL1-AF700 (DX9, Biolegend, San Diego, CA, USA), anti-NKG2A-PE-Cy7 (Z199, Beckman Coulter, Immunotech, Marseilles, France), anti-CD57-PB (HNK-1, Biolegend, San Diego, CA, USA), and anti-NKG2C-APC (REA205, Miltenyi Biotec, Paris, France). CD94-specific mAb was not included, as it was consistently co-expressed on NKG2A^+^ NK cells (20). Spillover and co-expression were taken into consideration. The experimental controls included unstained, single-stained, and fluorescence minus one controls. A lock template was created to acquire stained samples. All flow cytometry data were acquired using a unique FACSCanto II cytometer at our ISO 9001-certified research laboratory. BD FACSDiva CS&T Research Beads (BD Biosciences) were used prior to acquisition.

### Flow cytometry data analysis

The flow cytometry standard files were analyzed using Flowjo™ 10.6 software (LLC, Ashland, OR). They were then transferred to OMIQ data analysis software (OMIQ, Inc., Santa Clara, CA, USA). Dimensionality reductions were performed using the OMIQ software. After gating, subsampling of 50,000 NK cells per file was performed. Automated data cleaning was performed using PEACOQC to remove events with marginal values. The UMAP settings included random seeds. Subsequently, the FlowSOM settings included a run elbow meta-clustering on all 200 blood donors and seven features (KIR2DL1, NKp46, KIR2DL2/3, NKG2A, NKG2C, KIR3DL1, and CD57), resulting in 10 clusters. Metaclusters from the elbow method were visualized on a tree (data not shown).

### Hierarchical clustering analysis of NK cell subsets

Hierarchical clustering of the eight NK cell subsets of interest was performed following ward linkage using Genesis (31). The software is available at www.genome.tugraz.at.

### Statistical analyses

Categorical data were analyzed using the chi-square test, and univariate comparisons were performed using Student’s t-test. Comparisons between multiple groups were performed using one-way ANOVA. All statistical analyses were performed using the GraphPad Prism version 9.3.1 software (San Diego, CA). Statistical significance was set at P < 0.05.

## Results

### Strong association between KIR ligands

**Figure 1:**
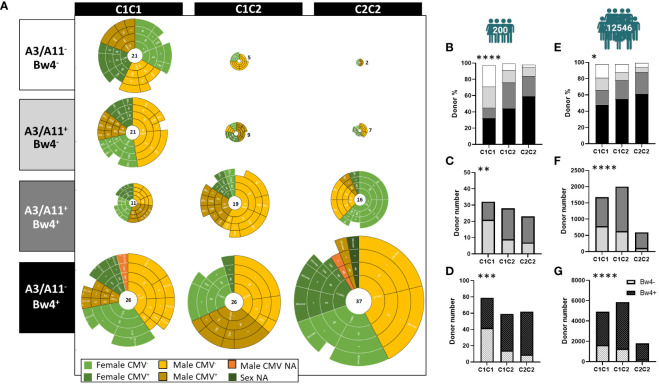
Strong association between KIR ligands. **(A)** Twelve sub-groups were determined from all volunteer blood donors based on C1, C2, A3/A11, and Bw4 KIR ligands, (N=200). Each pie groups female CMV^-^, female CMV^+^, male CMV^-^, and male CMV^+^ donors. NA (not available) was noted when CMV status, or sex was not advised. **(B)** The staked bar graphs show the frequencies of C1C1, C1C2, and C2C2 blood donors expressing KIR ligands: A3/A11^-^ Bw4^-^ (white), A3/A11^+^ Bw4^-^ (light gray), A3/A11^+^ Bw4^+^ (dark gray), and A3/A11^-^ Bw4^+^ (black) in the cohort of 200 healthy blood donors. **(C)** The staked bar graphs show the number of C1C1, C1C2, and C2C2 blood donors expressing A3/A11^+^ Bw4^-^ (light gray) or A3/A11^+^ Bw4^+^ (dark gray) in the cohort of 200 healthy blood donors. **(D)** The staked bar graphs show the number of C1C1, C1C2, and C2C2 blood donors expressing (crosshatch in black) or not (crosshatch in white) Bw4 KIR ligands in the cohort of 200 healthy blood donors. **(E)** The staked bar graphs show the frequencies of C1C1, C1C2, and C2C2 blood donors expressing KIR ligands: A3/A11^-^ Bw4^-^ (white), A3/A11^+^ Bw4^-^ (light gray), A3/A11^+^ Bw4^+^ (dark gray) and A3/A11^-^ Bw4^+^ (black) in the cohort of 12546 volunteer bone marrow donors. **(F)** The staked bar graphs show the number of C1C1, C1C2, and C2C2 blood donors expressing A3/A11^+^ Bw4^-^ (light gray), or A3/A11^+^ Bw4^+^ (dark gray) in the cohort of 12546 volunteer bone marrow donors. **(G)** The staked bar graphs show the number of C1C1, C1C2, and C2C2 blood donors expressing Bw4 KIR ligands (crosshatch in black) and those who do not (crosshatch in white) in the cohort of 12546 volunteer bone marrow donors. Chi-square contingency analysis used to compare different groups. Statistical significance was denoted as follows: *p<0.05, **p<0.01, ***p<0.001 and ****p<0.0001.

We established a cohort of 200 blood donors to investigate the effects of intrinsic and extrinsic markers on the phenotypic structure of the NK cell repertoire. We defined the three blood donor groups as C1C1 (n=79), C1C2 (n=59), and C2C2 (n=62). Thirty-three percent of blood donors were CMV seropositive. Moreover, this cohort included 44% of HLA-C homozygous donors and was thus not representative of Europeans. We first depicted this cohort after stratification along the C1, C2, A3/A11, and Bw4 KIR ligands. As a result, 12 groups were defined (**Figure 1A**). We also observed large disparities in the distribution of blood donors. Blood donors who did not express Bw4 (A3/A11^-^ or A3/A11^+^) were significantly more represented in C1C1 than in C1C2 or C2C2 (p<0.0001) (**Figure 1B**). The absence of Bw4 in A3/A11^+^ blood donors was significantly higher in C1C1 than in C1C2 and C2C2 blood donors (p<0.01) (**Figure 1C**). In contrast, C2^+^ (C1C2 and C2C2) blood donors frequently co-expressed Bw4 ligands (p<0.001) (**Figure 1D**). These observations were confirmed in a broad cohort of registered volunteer bone marrow donors (n=12546), representative of Europeans (**Figures 1E–G**). In the cohort of 200 blood donors, we observed more C2C2 females and C1C2 males in association with the Bw4 and A3/A11 ligands respectively (**Supplementary Figure 1A**). This association was not observed in the broader cohort of volunteer bone marrow donors. However, the distribution of Bw4^+^ HLA-A3/A11^-^ donors with seropositive CMV status was significantly different in females and males following treatment with HLA-C KIR ligands (p=0.01) (**Supplementary Figure 1A**), whereas the representation of C1C1, C1C2, and C2C2 was similar in both sexes. In the absence of Bw4, the donor distribution was similar according to sex or CMV status. In Bw4^+^ HLA-A3/A11^+^ donors, all C1C1 (C2^-^) males were CMV^+^, whereas 71% of C2^+^ were CMV^-^ (**Supplementary Figure 1B**). Taken together, these observations indicate a strong association between KIR ligands.

### KIR3DL1 subtly drives the phenotypic structure of the NK cell repertoire

**Figure 2:**
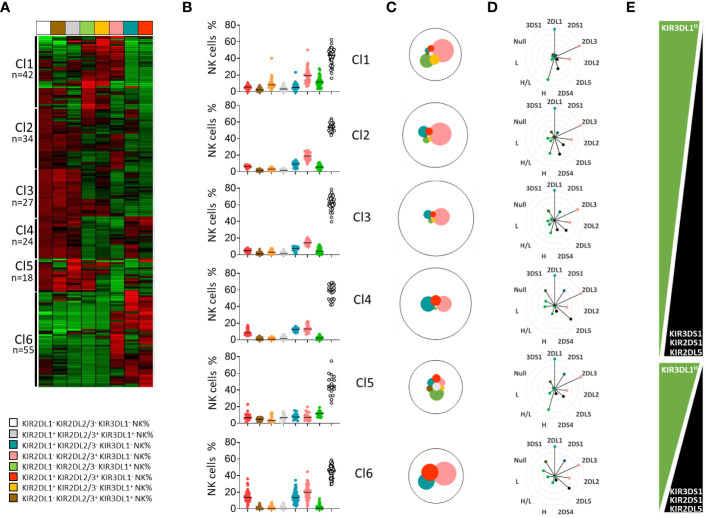
KIR3DL1 subtly drives the phenotypic structure of the NK cell repertoire. **(A)** A heatmap of NK cell subsets (n=8) is presented as the percentage of cells expressing a given phenotype. The figure displays the NK cell subsets for each of the 200 blood donors, with each subset indicated vertically and each donor indicated horizontally. **(B)** Dot plots represent the NK cell frequencies for the six clusters of blood donors defined by unsupervised clustering (Genesis^®^). **(C)** The mean frequency of the studied NK cell subsets in each cluster of blood donors is represented by pies, with the size of each pie being proportional to the NK cell frequency. **(D)** Alternative rose charts are used to represent the KIR (2DL1, 2DL2, 2DL3, 2DL5, 3DL1H (high), 3DL1H/L (high/low), 3DL1L (low), 3DL1Null, 3DS1, 2DS4, and 2DS1) genetic characteristics for each cluster of blood donors. **(E)** Graphic representation of the evolution of specific KIR (3DL1, 3DS1, 2DL5, and 2DS1) gene frequencies in the blood donor clusters.

To evaluate the effects of KIR and HLA genetic polymorphisms on the phenotypic structure of the NK cell repertoire, we determined, by flow cytometry, the frequency of KIR2DL1^+^, KIR2DL2/3^+^, KIR3DL1^+^, and four other subsets co-expressing these KIR and KIR^-^ NK subsets in blood donors (n=200). We further clustered these eight NK cell subsets from the cohort of 200 blood donors using unsupervised ward linkage clustering (Genesis^®^) (**Figure 2A**). Six clusters of blood donors with specific phenotypic profiles were identified (**Figure 2B**). The investigated NK cell subsets are illustrated according to their mean frequencies in all clusters of blood donors (**Figure 2C**). We observed broad diversity in the representation of these NK cell subsets, with dominance of the KIR^-^ NK cell subset. Genetic and allelic KIR typing was performed on all blood donors. The cohort comprised 37 blood donors with non-expressed KIR3DL1 (18.5%). For KIR3DL1, we deduced high (H), low (L), high/low (H/L), and null (null/-) expression of KIR3DL1 from both KIR3DL1 allelic typing and KIR3DL1 mean fluorescent intensity. Specific KIR genetic characteristics (i.e., KIR2DL1, 2DL2, 2DL3, 2DL5, 3DL1^H^, 3DL1^L^, 3DL1^H/L^, 3DL1^null/-^, 2DS4, 3DS1, and 2DS1 genes) were compiled for each cluster of blood donors to analyze their potential impact on the structure of the NK cell repertoire (**Figure 2D**). Interestingly, clusters 1 (n=42) and 2 (n=34) harbored the highest frequencies of the KIR2DS4 gene, whereas cluster 6 (n=55) harbored the lowest frequency of this gene, and its presence gradually decreased from cluster to cluster. This result was in accordance with the representation of KIR genotypes in all blood donor clusters (**Supplementary Figure 2A**). KIR3DL1 expression was high in clusters 1 and 5, in which blood donors mainly harbored KIR3DL1^H^ alleles (**Supplementary Figure 2B**). Cluster 1 had the highest frequency of the KIR3DL1^+^ KIR2DL2/3/S2^+^ NK cell subset, whereas cluster 5 (n=18) presented the highest frequency of the KIR3DL1^+^ KIR2DL1/S1^+^ KIR2DL2/3/S2^+^ NK cell subset. In contrast, the clusters 4 (n=24) and 6 (n=55) were characterized by a high frequency of KIR3DL1^null/-^, explaining a poor KIR3DL1 NK cell repertoire. In parallel with the decreased expression of KIR3DL1 from cluster to cluster, the frequency of KIR2DL5 and KIR2DS1 gradually increased from cluster 1 to cluster 4 and from cluster 5 to cluster 6 (**Figure 2E**). The presence of the KIR2DS1 gene was logically accompanied by increased expression of KIR2DL1/S1^+^ NK cells, as observed in clusters 4 and 6. Interestingly, clusters 1 and 5, which shared numerous genetic KIR characteristics, harbored different NK cell repertoire structures. Cluster 1 expressed a high frequency of KIR2DL2/3/S2^+^ NK cells, in contrast to cluster 5, in which NK cells co-expressing numerous KIR were well represented. The main difference between these two clusters can be explained by the differential KIR2DS4 and KIR2DL2 representation. Allelic KIR typing confirmed that the six donor clusters differed, particularly in the predominant KIR2DL1, KIR2DL2, KIR3DL1, and KIR2DS4 allele distributions (**Supplementary Figure 2B**). The HLA characteristics of each cluster in terms of KIR ligands (A3/A11, C1, C2, and Bw4) did not explain the main differences in the phenotypic structure of the KIR NK cell repertoires (**Supplementary Figure 2C**). Finally, we investigated the potential impact of age (<50 or >50 years), sex (male or female), and CMV status (**Supplementary Figure 2D**) on the phenotypic structure of the NK cell repertoire. No features seemed to be involved in driving the phenotypic KIR NK cell structure. Altogether, our observations show the importance of the level of expression of the KIR3DL1 allele-encoded product in the phenotypic structure of the KIR NK cell repertoire.

### KIR2DL2 and C1/C2 KIR ligands drive the phenotypic structure of the KIR2D^+^ NK cell repertoire

**Figure 3:**
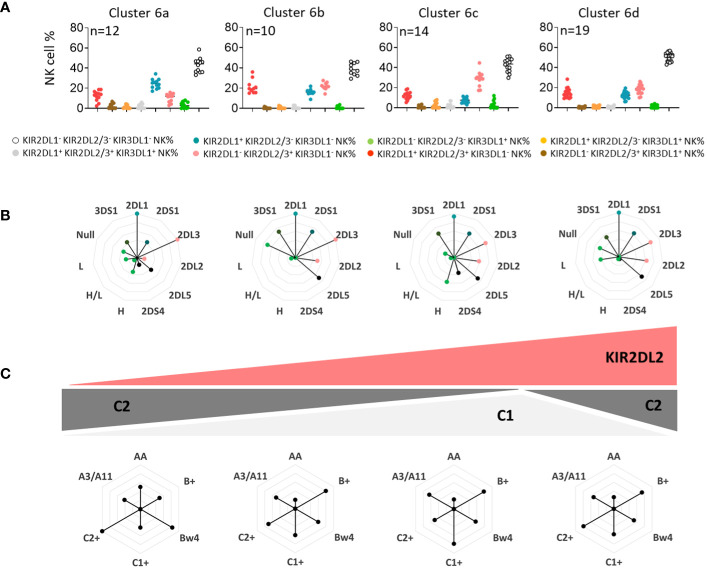
KIR2DL2 and C1/C2 KIR ligands drive the phenotypic structure of the KIR2D^+^ NK cell repertoire. **(A)** Dot plots represent the NK cell frequencies for the four sub-clusters 6 (6a, 6b, 6c, and 6d). **(B)** The genetic characteristics of KIR (KIR3DL1, 2DL2, 3DS1, 2DS4, and 2DS1) for each sub-cluster of blood donors are represented in alternative rose charts. **(C)** The alternative rose charts represent the KIR ligands (C1, C2, Bw4, and A3/A11) and KIR genotype (AA, B^+^) characteristics for each sub-cluster of blood donors. Graphic representation of the evolution of the KIR2DL2 gene and C1/C2 ligands in sub-clusters of blood donors is shown.

Using unsupervised clustering, we determined six main clusters among blood donors. Sub-clustering, particularly for cluster 6, which was marked by the absence of KIR3DL1 expression and an important number of blood donors (n=55), was further conducted to determine the secondary influence of particular KIR genes and/or ligands. Thus, four sub-clusters of cluster 6 (a, b, c, and d) were identified and compared (**Figure 3A**). Interestingly, the main difference was in the frequency of KIR2DL1/S1^+^ and KIR2DL2/3/S2^+^ NK cell subsets, with a high frequency of KIR2DL1/S1^+^ NK cells in cluster 6a, which declined from cluster to cluster until cluster 6c. This decreased representation was directly linked to KIR2DL2, whose frequency increased from cluster 6a to cluster 6c (**Figure 3B**). Moreover, the C2 ligands were highly represented in cluster 6a, and this representation declined in cluster 6c (**Figure 3C**). Radar charts of genetic KIR characteristics were similar for clusters 6c and 6d; however, cluster 6d blood donors showed significant expression of C2 ligands. Altogether, KIR2DL2 and C1/C2 KIR ligands were subtly interlinked to drive the phenotypic structure of the KIR2D^+^ NK cell repertoire.

### The frequency of educated NK cells depends on the nature and the number of KIR ligands and increases with age and CMV infection

**Figure 4:**
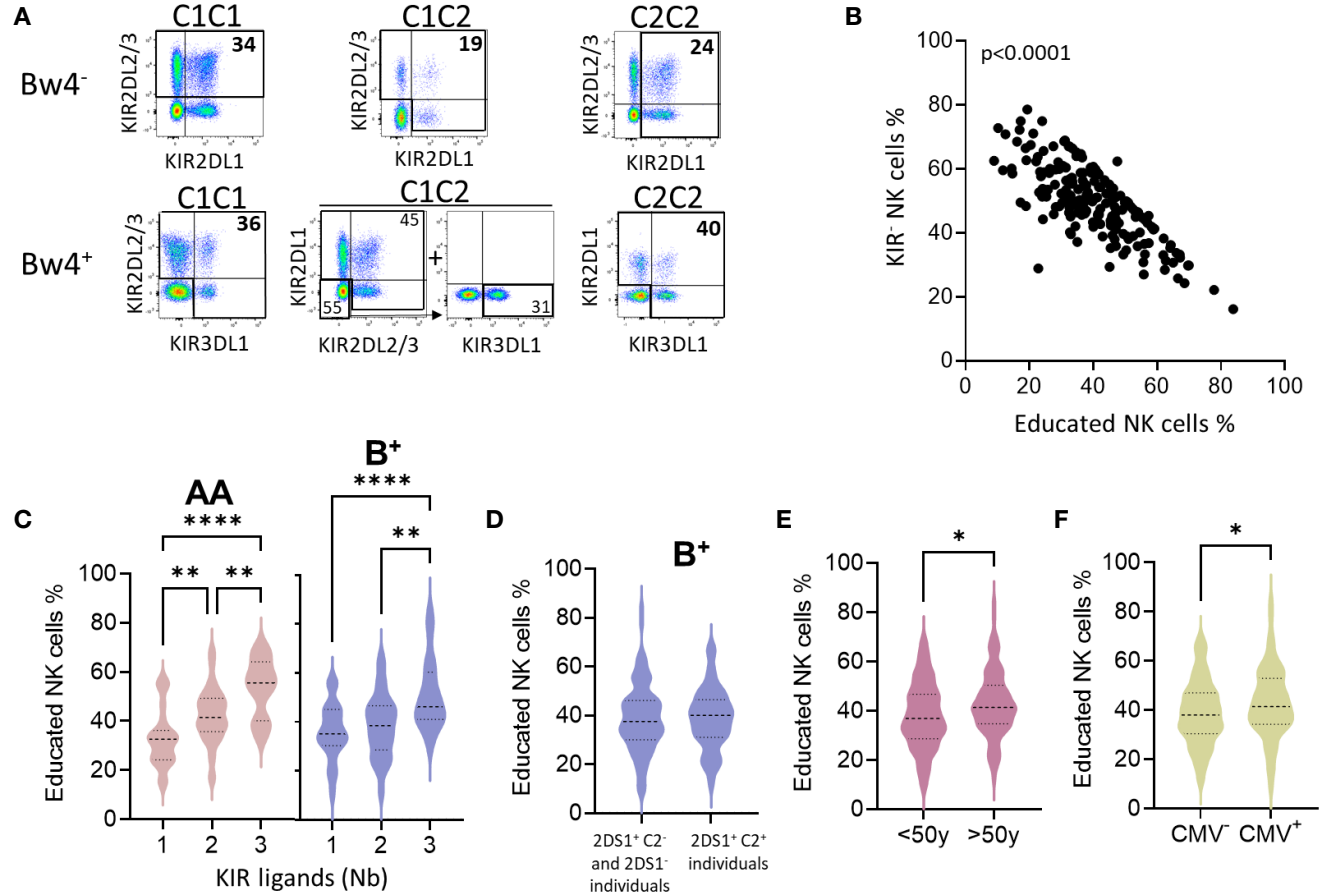
The frequency of educated NK cells depends on the nature and the number of KIR ligands and increases with age and CMV infection. **(A)** The gating strategy of educated NK cells is illustrated for C1, C2, and Bw4 as KIR ligands. **(B)** Dot plots show the correlation between KIR^-^ NK cell frequencies and educated NK cell frequencies in all blood donors. **(C)** Violins present educated NK cell frequencies based on the number of KIR ligands (1 to 3) in AA (n=77), and B^+^ (n=123) blood donors. Bw4 and C2 KIR ligands were not considered for blood donors who do not express KIR3DL1 (KIR3DL1^null^ or KIR3DL1^-^) and KIR2DL1 respectively. Group comparisons were conducted using one-way ANOVA. **(D)** Violins show educated NK cell frequencies in a group of individuals without a functional KIR2DS1 receptor (KIR2DS1^-^ and KIR2DS1^+^ C2^-^) compared to those with a functional KIR2DS1 receptor (KIR2DS1^+^ C2^+^). **(E)** Violins show educated NK cell frequencies in young (<50y) and old (>50y) blood donors, and **(F)** in CMV^-^ and CMV^+^ blood donors. Univariate comparisons were performed using the Student’s t-test. Statistical significance was denoted as follows: *p<0.05, **p<0.01 and ****p<0.0001.

We further evaluated the frequency of educated KIR^+^ NK cells by considering C1C2 and Bw4 KIR ligands, following the gating strategy illustrated for all Bw4^-^ and Bw4^+^ profiles (C1C1, C1C2, and C2C2) (**Figure 4A**). The frequency of educated KIR-NK cells was inversely correlated with that of KIR^-^ NK cells (**Figure 4B**). The frequency of educated NK cells was similar between AA and B^+^ blood donors (data not shown). However, the number of KIR ligands (C1, C2, and Bw4) increased the frequency of educated NK cells (**Figure 4C**). HLA-A3/A11 does not constitute a ligand that appears to contribute to NK cell education (data not shown), as previously reported (32). Although not significant, the frequency of educated KIR NK cells was higher in AA than in B^+^ blood donors with the three KIR ligands. In B^+^ blood donors, activating KIR genes can negatively modulate NK cell education, as previously described (33). We further evaluated the effect of the well-known KIR2DS1 gene and found that it did not affect the frequency of the pool of educated NK cells in B^+^ blood donors (**Figure 4D**). Sex did not affect the number of educated NK cells (data not shown). Finally, the frequency of educated NK cells significantly increased with age (**Figure 4E**) and CMV status (**Figure 4F**). Taken together, these data show that C1-, C2-, and Bw4-rich environments contribute to the mounting of a large pool of functionally educated NK cells. Moreover, CMV infection and advanced age boost the pool of educated NK cells.

### The nature of KIR ligands influences the maturation profile of NK cells

**Figure 5:**
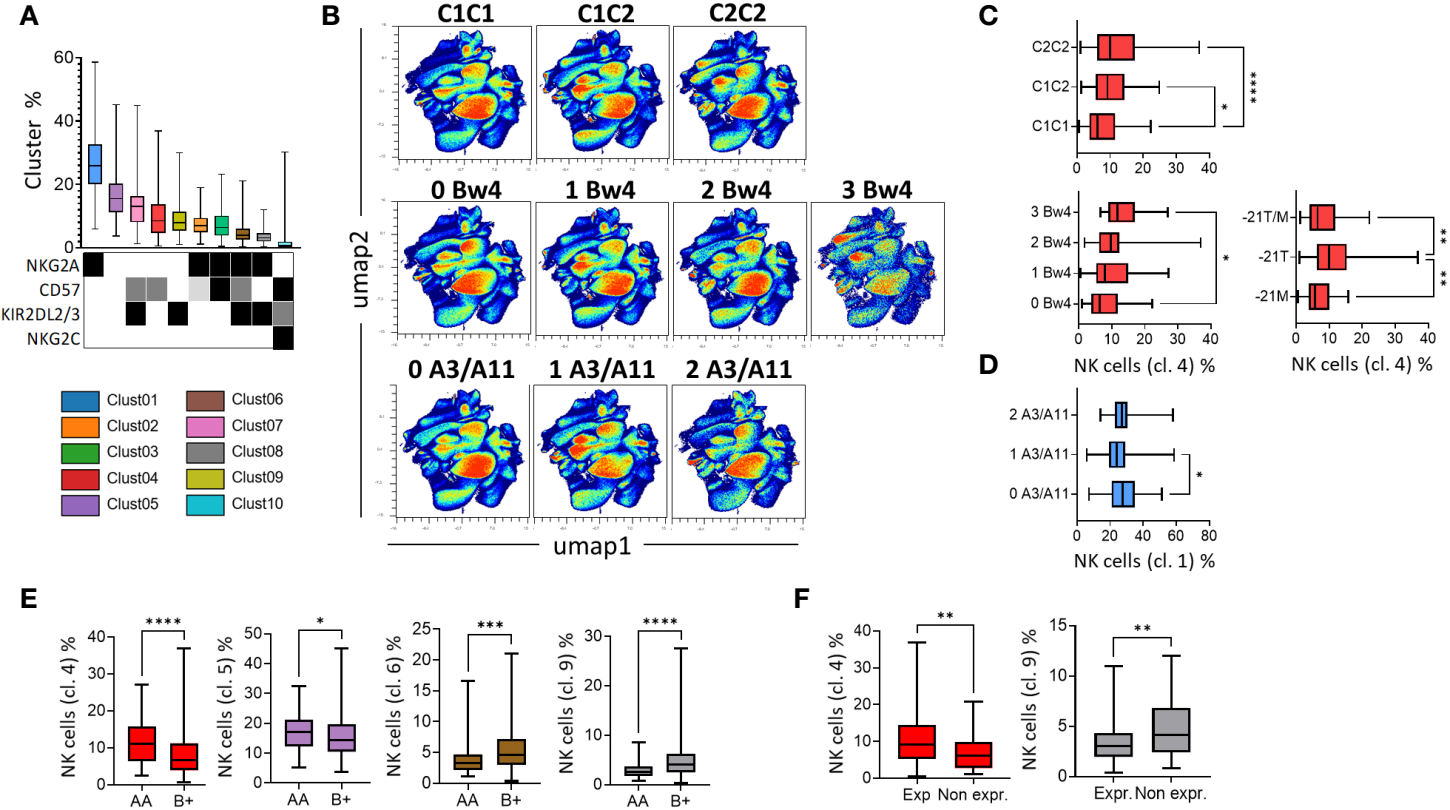
The nature of KIR ligands and KIR genotypes influence the differentiation profile of NK cells. **(A)** Box-and-whisker plots represent the mean of the 10 NK cell clusters, ordered from the best represented to the lowest. The expression of NKG2A, CD57, KIR2DL2/3, and NKG2C is indicated by a square with different colors denoting the level of expression (low in light grey, intermediate in grey, and high in black). **(B)** High-dimensional data analysis of NK cells from healthy donors displayed into two UMAP dimensions. The density plots show concatenated events from all of the indicated samples. UMAP density plots were used to visualize the clustering results of 200 blood donors based on KIR2DL1/S1, KIR2DL2/3/S2, KIR3DL1, NKG2A, CD57, and NKG2C expression on NK cells following KIR ligand (C1, C2, Bw4, and A3/A11) environment. **(C)** Box-and-whisker plots represent the mean frequency of NKG2A^-^ KIR^-^ CD57^+^ NK cells (cluster 4) in blood donors following KIR ligand (C1, C2, and Bw4) and -21T/M, -21T, or -21M motifs. **(D)** Box-and-whisker plots represent the mean frequency of KIR^-^ NKG2A^+^ CD57^-^ (cluster 1) in blood donors harboring 0, 1, or 2 HLA-A3/A11 ligands. **(E)** Box-and-whisker plots represent the mean frequency of NKG2A^-^ KIR^-^ CD57^+^ (cluster 4), NKG2A^-^ KIR^-^ CD57^-^ (cluster 5), NKG2A^+^ KIR^+^ CD57^+^ (cluster 6) and NKG2A^+^ KIR^+^ CD57^-^ (cluster 8) in AA versus B^+^ KIR genotyped blood donors. **(F)** Box-and-whisker plots represent the mean frequency of NKG2A^-^ KIR^-^ CD57^+^ (cluster 4) and NKG2A^+^ KIR^+^ CD57^-^ NK cells (cluster 8) in KIR3DL1^exp^ versus 3DL1^null/-^ blood donors. Univariate comparisons were performed using the Student’s t-test, and group comparisons were performed using one-way ANOVA. Statistical significance was denoted as follows: *p<0.05, **p<0.01, ***p<0.001 and ****p<0.0001.

In parallel with the supervised analysis, we performed an unsupervised analysis using OMIQ software based on 200 blood donors with the NK cell phenotype, including NKp46 (NK cells), KIR2DL1/S1, KIR2DL2/3/S2, KIR3DL1, NKG2A, NKG2C, and CD57. Ten clusters of NK cell subsets were identified. The NK cell subsets were classified according to their frequency in all blood donors (**Figure 5A**). These clusters were characterized by differential expression of NKG2A, CD57, KIR2DL2/3/S2, and NKG2C. The undifferentiated NKG2A^+^ KIR^-^ CD57^-^ NK cell subset was the most abundant subset. In contrast, the memory NKG2C^+^ KIR^+^ CD57^+^ NK cells were less abundant (**Figure 5A**). Notably, the range of NK cell frequencies was particularly important for all blood donors, underlying the broad diversity of the NK cell repertoire. UMAP profiles as a function of the number of KIR ligands are presented in **Figure 5B**. We observed an increased distribution of cluster 4, which was characterized by the expression of CD57 only in C2^+^ donors (C1C2 and C2C2) and in blood donors with a high number of Bw4 ligands (**Figure 5C**). Interestingly, the -21T motif, mainly displayed in the Bw4 and C2 molecules, was associated with a higher frequency of NKG2A^-^ KIR^-^ CD57^+^ NK cells (cluster 4). Finally, the immature NKG2A^+^ NK cell subset (cluster 1) was more abundant in the absence of HLA-A3/A11 ligands (**Figure 5D**). These data show the main influence of KIR ligands on the maturation profile of NK cells, with a high distribution of the NKG2A^-^ KIR^-^ CD57^+^ NK cell subset in the C2 and Bw4 environments, in contrast to the high distribution of the immature NKG2A^+^ NK cell subset in the absence of HLA-A3/A11 ligands.

### KIR B^+^ and AA genotypes respectively drive a high distribution of KIR2DL2/3/S2^+^ NKG2A^+^ and KIR2DL2/3/S2^-^ NKG2A^-^ NK cell subsets

We investigated the influence of KIR genotypes on the phenotypic structure of the NK cell repertoire. Cluster 4 (NKG2A^-^ KIR^-^ CD57^+^) and, to a lesser extent, cluster 5 (NKG2A^-^ KIR^-^ CD57^-^) were more abundant in AA (n=76) blood donors than in B^+^ (n=124) blood donors (**Figure 5E**). In contrast, clusters 6 and 8 (NKG2A^+^ KIR^+^ CD57^+/-^) were more represented in B^+^ compared to AA blood donors. KIR3DL1 harbors a broad allelic polymorphism, and some KIR3DL1 allele-encoded products are not expressed on NK cell surfaces. These variants are associated with the B^+^ KIR genotypes. Blood donors expressing KIR3DL1 displayed a higher frequency of CD57^+^ NK cells (cluster 4), whereas those who did not express KIR3DL1 displayed a higher frequency of the NKG2A^+^ KIR2DL2/3/S2^+^ CD57^-^ NK cell subset (cluster 8) (**Figure 5F**). Altogether, our data showed that KIR B^+^ and AA genotypes respectively drive a high distribution of KIR2DL2/3/S2^+^ NKG2A^+^ and KIR2DL2/3/S2^-^ NKG2A^-^ NK cell subsets.

### In the context of aging and CMV status, CD57^+^ NK cells take over from the immature NKG2A^+^ NK cells and are more prevalent in males

**Figure 6:**
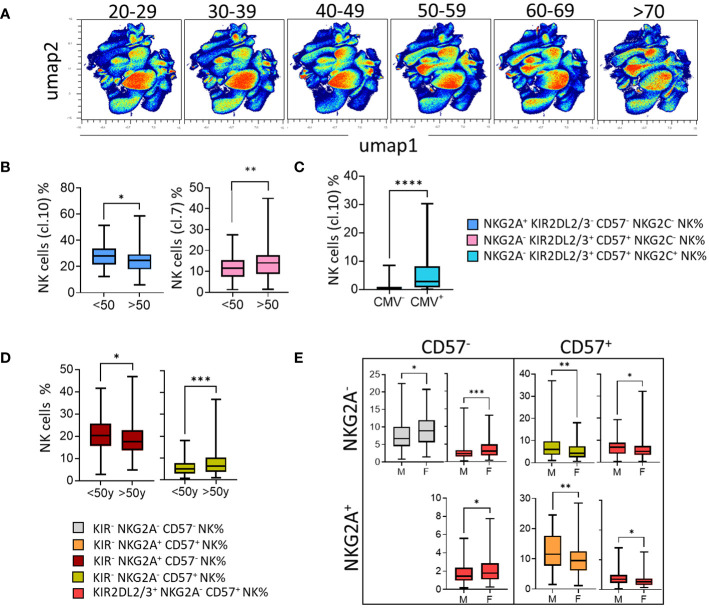
In the context of aging and CMV status, CD57^+^ NK cells take over from the immature NKG2A^+^ NK cells and are more prevalent in males. **(A)** High-dimensional data analysis of NK cells from healthy donors displayed into two UMAP dimensions. The density plots show concatenated events from all of the indicated samples. UMAP density plots visualizing the results of clustering 200 blood donors based on 10-year age brackets (20-29, 30-39, 40-49, 50-59, 60-69, and >70 years). **(B)** Box-and-whisker plots show the mean frequency of NKG2A^+^ KIR^-^ CD57^-^ NK cells (cluster 1) and NKG2A^-^ KIR^+^ CD57^+^ NK cells (cluster 7) in young (<50y) and old (>50y) blood donors. **(C)** Box-and-whisker plots show the mean frequency of NKG2C^+^ KIR^+^ CD57^+^ NK cells (cluster 10) in CMV^-^ versus CMV^+^ blood donors. **(D)** The NK cell subsets were identified by the expression of KIR, NKG2A, and CD57. The mean frequency of KIR^-^ NKG2A^+^ CD57^-^ NK and KIR^-^ NKG2A^-^ CD57^+^ NK cell subsets in young (<50y) and old (>50y) blood donors is shown in box-and-whisker plots. **(E)** Box-and-whisker plots represent the mean frequency of NK cells expressing or not CD57 in co-expression or not with NKG2A in males and females. Univariate comparisons were performed using the Student’s t-test. Statistical significance was denoted as follows: *p<0.05, **p<0.01, ***p<0.001 and ****p<0.0001.

To complete our study, we further investigated the impact of age and sex on the phenotypic structure of the NK cell repertoire. Using an unsupervised approach (UMAP), the distribution of NK cells was presented as density scatterplots in 10-year age brackets (**Figure 6A**). Immature NKG2A^+^ KIR^-^ CD57^-^ NK cells (cluster 1) were significantly more represented in younger blood donors (<50 years) (**Figure 6B**). Moreover, mature NKG2A^-^ KIR2DL2/3/S2^+^ CD57^+^ NK cells (cluster 7) were significantly more abundant in older blood donors (>50 years) (**Figure 6B**). As expected, the KIR2DL2/3/S2^+^ NKG2C^+^ CD57^high^ NK cell subset was significantly associated with CMV seropositivity (**Figure 6C**). Interestingly, the absence of KIR2DL5 was significantly associated with better representation of this memory NK cell subset (data not shown).

In parallel with this unsupervised analysis, we performed a supervised analysis of 32 NK cell subsets determined from NKG2A, CD57, and KIR (KIR2DL1, KIR2DL2/3, and KIR3DL1). Among the more represented NK cell subsets (mean threshold > 1%) from all identified NK cell subsets (n=32), KIR^-^ NK cells were predominant, particularly KIR^-^ CD57^-^ NKG2A^+^ NK cells (data not shown). KIR^+^ NK cell subsets preferentially express KIR2DL2/3/S2 and KIR2DL1/S1, and to a lesser extent, KIR3DL1. Using this approach, we showed that the KIR2DL1/S1^+^ NKG2A^-^ CD57^+^ NK subset was more frequent in blood donors displaying C2 ligands, the AA genotype, and the Cen AA motif, characterized by the absence of KIR2DL2 (**Supplementary Figure 3A**). In contrast, KIR2DL2/3/S2^+^ NKG2A^-^ CD57^+^ NK cell subset was more frequent in blood donors displaying C1 ligands (**Supplementary Figure 3A**). The KIR3DL1^+^ NKG2A^-^ CD57^+^ NK cell subset was more frequent in blood donors displaying the KIR AA genotype and Tel AA motif, while the KIR3DL1^+^ KIR2DL2/3/S2^+/-^ NKG2A^-^ CD57^+^ NK cell subset was more frequent in blood donors displaying the KIR AA genotype (**Supplementary Figure 3A**). Similarly, the KIR^-^ NKG2A^+^ CD57^-^ NK cell subset, the most frequent subset, was more frequent in blood donors displaying KIR2DL5 and those without the HLA-A3/A11 KIR ligand (**Supplementary Figure 3B**). Immature KIR^-^ NKG2A^+^ CD57^-^ NK cell subsets were higher in younger blood donors (<50 years) than in older blood donors (>50 years), which is in accordance with our results obtained using an unsupervised strategy (**Figure 6D**). The frequency of CD57^-^ NK cell subsets (NKG2A^-^ KIR^-^ CD57^-^ and NKG2A^+/-^ KIR2DL2/3/S2^+^ CD57^-^) was higher in females than in males (**Figure 6E**). In contrast, the frequency of CD57^+^ NK cell subsets (NKG2A^+/-^ KIR2DL2/3/S2^+/-^ CD57^+^) was higher in males than in females. Altogether, our data show that the rules that guide the phenotypic structure of the NK cell repertoire integrate not only KIR and KIR ligands but also age, sex, and CMV status.

## Discussion

Numerous association and linkage studies have highlighted the pivotal role of the HLA locus in the genetic susceptibility to a multitude of autoimmune diseases. However, linkage disequilibrium may result from population mixture or genetic drift facilitated by natural selection. In this study, we investigated the distribution of HLA class I molecular groups as ligands for KIRs. We observed a more frequent association between C2 and Bw4 ligands. In contrast, blood donors without Bw4 ligands preferentially exhibited C1C1. In the presence of Bw4, C1C1 blood donors did not express HLA-A3/A11 ligands. Notably, the similar frequencies of the C1C1, C1C2, and C2C2 individuals arbitrarily defined in this study are not representative of Europeans. C2 and Bw4 show strong linkage disequilibrium in Europeans (34), which has not been observed in Japanese individuals (35). The C2 and Bw4 tandem should mobilize the KIR2DL1 and KIR3DL1 NK cell subsets, whereas the HLA-A3/A11 and C1 tandem should mobilize the KIR2DL2/3 and KIR3DL2 NK cell subsets to detect a variety of possible threats.

The phenotypic structure of the KIR NK repertoire appears early in life, and only the frequency of this repertoire increases until adulthood. Although similar overall, the inhibitory NK cell receptor repertoires of twins exhibit some differences, which might be explained by environmental influences (20). Consistent with previous studies, we observed that the presence of cognate HLA class I ligands increased the frequency of NK-expressing inhibitory KIR (13, 35). Notably, KIR2DL2/3 and KIR3DL1/S1 are present in most KIR haplotypes (6). KIR3DL1/S1 harbors a large allelic polymorphism that significantly affects KIR3DL1 expression. It is first expressed in NK cells before KIR2DL2/3 and KIR2DL1 are acquired in the last step (36). We previously observed a constant proportion of KIR3DL1^high^ NK cells expressing KIR2DL1/2/3 and 2DS1/2 receptors, suggesting the subsequent acquisition of KIR2D by KIR3DL1^high^ NK cells (37). Moreover, in accordance with this, we showed that nearly 50% of KIR3DL1^+^ NK cells from umbilical cord blood express KIR2D receptors in early life (38). Furthermore, KIR2DL2 is expressed a second time before KIR2DL1, and KIR2DL2 competes with KIR2DL1 for C2 ligands (36). Thus, based on the levels of KIR3DL1 expression and the presence of KIR2DL2, we predicted the approximate configuration of the KIR NK cell repertoire.

We found that the frequency of educated KIR NK cells was inversely correlated with that of KIR^-^ NK cells. Interestingly, we previously showed that the frequency of KIR^-^ NK cells inversely correlates with rituximab-dependent CD107a^+^ NK cells, suggesting that this frequency is a good prognostic marker of rituximab response in the absence of genetic KIR and HLA typing (39). Similarly, the frequency of the KIR^-^ population can help to estimate the pool of educated NK cells in the absence of KIR and HLA genetic backgrounds.

NKG2A^+^ NK cells constitute the most prevalent NK cell population, which express none of the cell surface markers and co-express KIR2DL2/3 and CD57. We did not observe the prevalence of NKG2A^+^ NK cells in the -21M motif-enriched HLA class I environment, as previously reported (34). We observed a strong linkage disequilibrium between C2 and Bw4 ligands that share the -21T motif. This -21T motif, as well as Bw4 (essentially HLA-B) and C2 KIR ligands, favors CD57^+^ NK cells. We suggest that all HLA-A molecules supply -21M peptides to HLA-E, maintaining CD94/NKG2A recognition. The absence of HLA-A3/A11 ligands favors the NKG2A^+^ NK cell population. NKG2A is more frequently expressed with KIR3DL2 than with KIR2DL and confers full functional competence on KIR3DL2^+^ NK cells (32). However, in the absence of documentation on KIR3DL2 expression, it is difficult to explain this observation in relation to KIR3DL2 expression. Moreover, NKG2A expression is inversely correlated with the number of co-expressed KIR genes, as observed in a cohort of 31 AA KIR-genotyped blood donors (32). We confirmed this with the finding that KIR B^+^ and AA genotypes respectively drive a high distribution of KIR^+^ NKG2A^+^ and KIR^-^ NKG2A^-^ NK cell subsets.

We observed the evolution of the NK cell repertoire, predominantly marked by less-differentiated NKG2A^+^ NK cell subsets in young people, to differentiated CD57^+^ NK cell subsets enriched in the NK cell repertoire in older people. These findings are in accordance with a larger pool of CD57^+^ lymphocytes with age and the knowledge that CD57^+^ cells harbor short telomeres and constitute a senescent-like phenotype (40). The number of NK cells increases over time, with clear differences according to sex for certain age ranges, along with a higher NK cell frequency in males than in females between the ages of 40–70 years (41, 42). However, to our knowledge, few studies have highlighted the influence of sex on the NK cell repertoire. We observed that the frequencies of CD57^-^ NK cell subsets (NKG2A^-^ KIR^-^ CD57^-^ and NKG2A^+/-^ KIR2DL2/3/S2^+^ CD57^-^) and CD57^+^ NK cell subsets (NKG2A^+/-^ KIR2DL2/3/S2^+/-^ CD57^+^) were associated with females and males, respectively. Finally, immune history shapes the NK repertoire, particularly CMV, for which memory-like NKG2C^+^ NK cell subsets constitute a strong signature. CMV significantly drives educated KIR NK cells to a mature CD57 state. However, their influence is limited to the formation of a global NK cell compartment.

Other parameters not included in this study may have modulated the structure of the NK cell repertoire. Environmental influences, such as smoking, exercise (43), diet, and microbiome, can shape the NK cell repertoire. For example, it has been shown that active smokers show a decreased number of NK cells (18). CD57^+^ and NKG2C^+^ NK cells constitute a large proportion of the NK cells in smokers (44). Furthermore, a study from a broader cohort should help replicate these findings and define the hierarchical influence of all factors more subtly. Moreover, the potential influences of HLA and sex on CMV infection require further investigation to gain an accurate and in-depth understanding of their functional interactions. Based on our findings, investigations in other populations, geographic locations, and ethnicities should be of particular interest because of the variability in KIR and HLA organizations worldwide. Similarly, the functional aspects of metabolism were not investigated in this study; however, they deserve attention. Indeed, high mTOR activity is a hallmark of reactive NK cells and amplifies early signaling by activating receptors (45). A large variation in mTORC1 activity has been identified at the donor and subset levels, suggesting the potential impact of HLA polymorphisms on NK cell receptors (46).

Overall, the number of mature CD57^+^ NK cells increased with age at the expense of immature NKG2A^+^ NK cells. Remarkably, sex contributes to modulating this shaping, as males present a more mature CD57^+^ NK repertoire than females. Intrinsic markers, such as KIR and HLA immunogenetics, drive the early formation of the KIR NK cell repertoire, and immune history during childhood contributes to the molding of the repertoire. These modulations persist throughout life, contributing to the maturation of repertoires with age, and they are influenced by sex. Functional investigations of NK cell subsets should be coupled with transcriptomic analyses to gain insights into NK cell biology. Accurate knowledge of the rules governing the formation of the NK cell repertoire and its modulation is essential for better selection of hematopoietic stem cell donors and differentiation of allogeneic NK cells from healthy donors for cell engineering in NK cell-based immunotherapy approaches.

## Data availability statement

The raw data supporting the conclusions of this article will be made available by the authors without undue reservation.

## Ethics statement

The studies involving humans were approved by Authorization for the preparation and conservation of biocollections (AC-2021-4397) was provided by the French Research Minister. The studies were conducted in accordance with the local legislation and institutional requirements. The participants provided their written informed consent to participate in this study.

## Author contributions

EF: Writing – review & editing, Formal analysis, Investigation, Software, Visualization. GD: Data curation, Formal analysis, Methodology, Resources, Software, Writing – review & editing. CW: Data curation, Formal analysis, Methodology, Resources, Software, Writing – review & editing. NL: Data curation, Formal analysis, Methodology, Resources, Software, Writing – review & editing. PS: Formal analysis, Software, Writing – review & editing. LA: Resources, Writing – review & editing. AW: Resources, Writing – review & editing. KGe: Methodology, Resources, Writing – review & editing. KGa: Data curation, Formal analysis, Funding acquisition, Methodology, Resources, Validation, Writing – review & editing. CR: Conceptualization, Data curation, Formal analysis, Funding acquisition, Methodology, Project administration, Supervision, Validation, Visualization, Writing – original draft, Writing – review & editing.
