# Supplementary material for: Multifactorial determinants of NK cell repertoire organization: insights into age, sex, KIR genotype, HLA typing, and CMV influence
Source: Front Immunol. 2024 Apr 26;15:1389358. doi: 10.3389/fimmu.2024.1389358 (PMC11082329; doi:10.3389/fimmu.2024.1389358)
Supplement: Supplementary file 1 [file DataSheet_1.pdf]

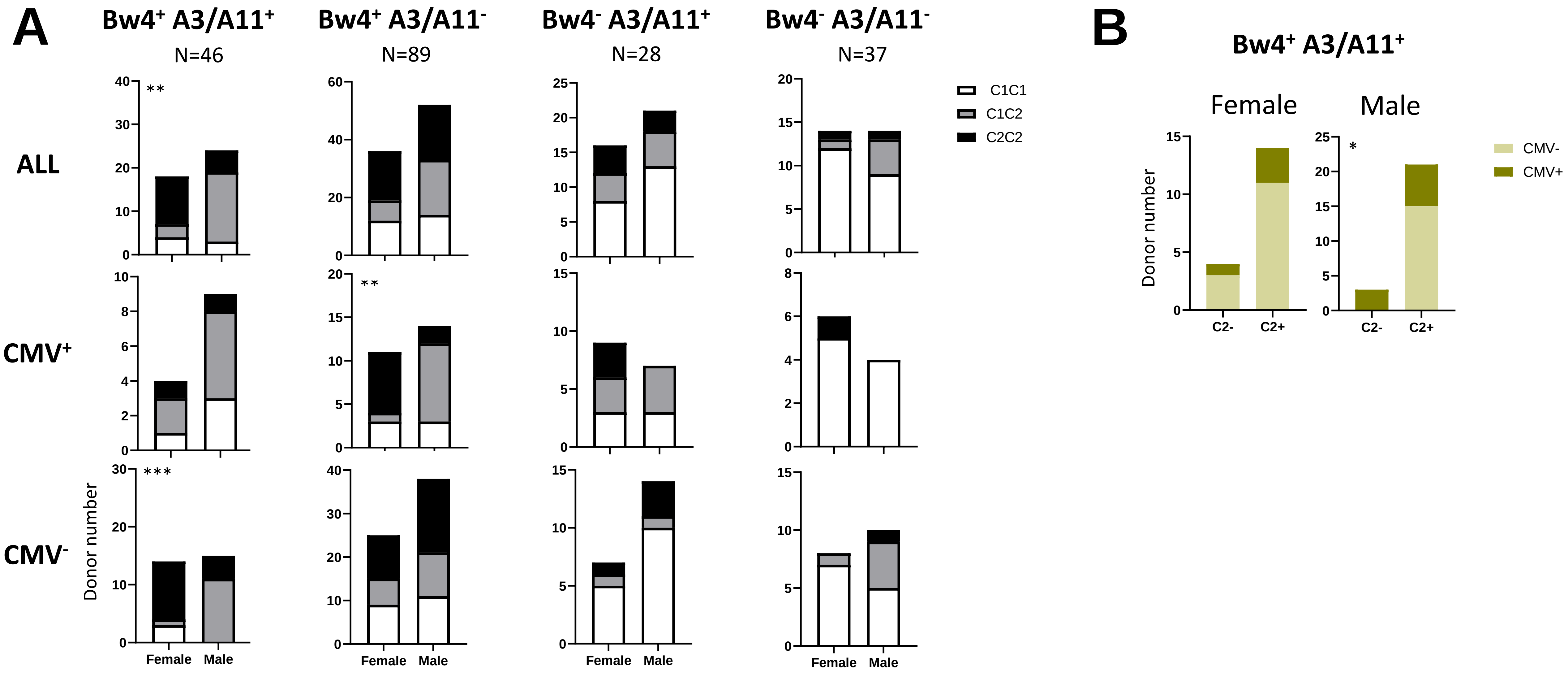

Supplemental figure 1. **A.** Distribution of Bw4⁺ HLA-A3/A11⁺, Bw4⁺ HLA-A3/A11⁻, Bw4⁻ HLA-A3/A11⁺ and Bw4⁻ HLA-A3/A11⁻ blood donors depending on HLA-C environment (C1C1, C1C2 and C2C2) following gender (female and male) and CMV status. **B.** Distribution of Bw4⁺ HLA-A3/A11⁺ blood donors in females and males following HLA-C environment (C2- vs C2+) and CMV status. Chi-square contingency analysis used to compare the different groups. *$p<0.05$, **$p<0.01$, ***$p<0.001$.

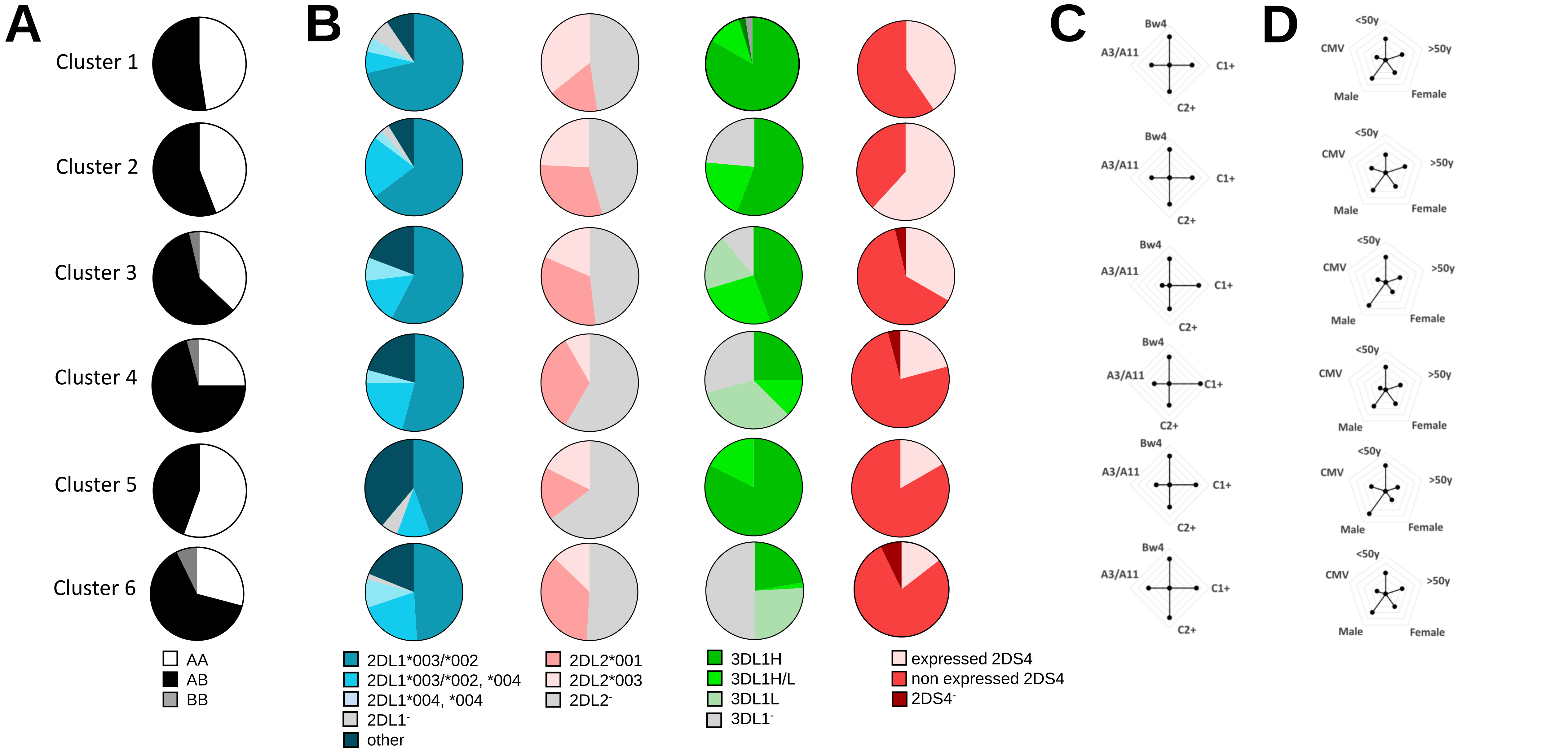

Supplemental figure 2. Distribution of blood donors in clusters defined by unsupervised clustering (Genesis®) following KIR genotypes, KIR alleles, KIR ligands, sex, age and CMV status. **A**. Pies representing the mean frequency of blood donors having AA, AB and BB KIR genotypes in the 6 clusters of blood donors. **B**. Pies representing the mean frequency of blood donors following KIR2DL1 (2DL1*002,*003 and *004), or KIR2DL2 (*001 and *003) predominant alleles. Pies representing the mean frequency of blood donors having 2 KIR3DL1 alleles leading a high membrane expression (3DL1H: 3DL1*001, *002, *008, *015), a low membrane expression (3DL1L: 3DL1*005, *007), one low and one high KIR3DL1 allele (3DL1H/L) and no KIR3DL1 expression (3DL1$^-$ or 3DL1*004^{null}), and finally pies representing the mean frequency of blood donors following expressed (2DS4*001) and non expressed KIR2DS4 (2DS4*003, *006) predominant alleles. **C**. The frequency of blood donors harboring KIR ligands (HLA-A3/A11, Bw4, C1 and C2) is represented in alternative rose charts for each cluster of blood donors. **D**. The frequency of blood donors following their age, sex and CMV seropositive status is represented in alternative rose charts for each cluster of blood donors.

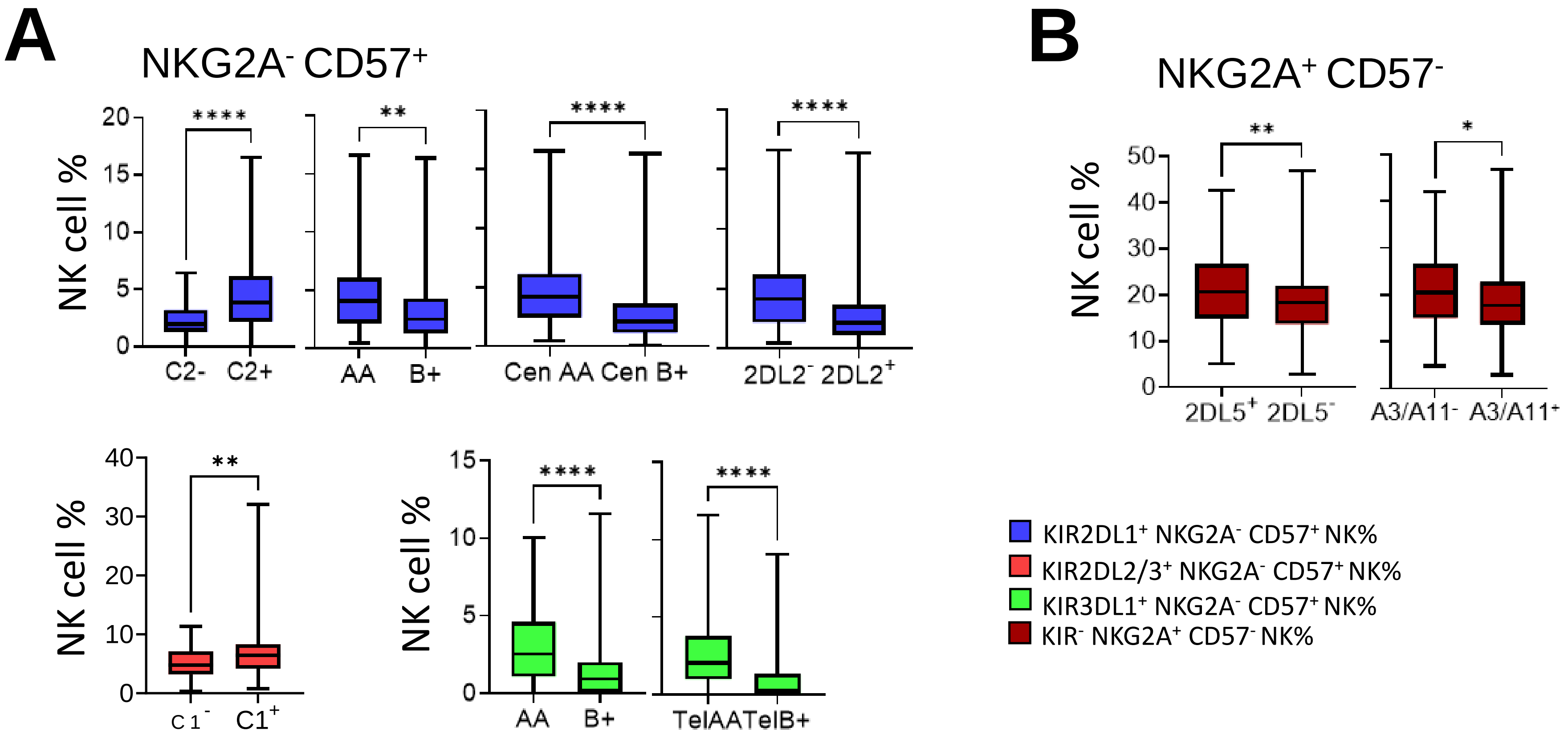

Supplemental figure 3. A. Frequency of NKG2A$^-$ CD57$^+$ NK cells expressing KIR2DL1, KIR2DL3 and KIR3DL1 following KIR ligands and KIR genotypes. **B.** Frequency of NKG2A$^+$ CD57$^-$ NK cells following the presence or absence of KIR2DL5 gene and HLA-A3/A11 environment. Univariate comparisons were performed by the Student's t-test. *p<0.05, **p<0.01 and ****p<0.0001.
